# Clinical Presentation and Outcome of Multiple Rare Earth Magnet Ingestions in Children of Qatar. A Single-Center Experience

**DOI:** 10.5339/qmj.2023.9

**Published:** 2023-02-20

**Authors:** Abdullah Khan, Yazeed Eldos, Khalid Alansari

**Affiliations:** Sidra Medical and Research Center. E-mail: abdullahkhan120@gmail.com ORCID ID is 0000-0003-4314-5202

**Keywords:** Rare Earth Magnets, Intestinal perforation, Children

## Abstract

Introduction: Rare earth magnets are powerful magnets that can have several negative effects if ingested. The goal of our study is to describe the result of multiple rare earth magnets ingested by children in Qatar.

Materials and methods: This is observational research. We conducted a retrospective chart review and descriptive analysis of all cases of multiple rare earth magnetic ingestion that werepresented to the Emergency Department of Sidra Medicine between January 2018 and July 2022. We obtained an exemption for this study from our institutional review board (IRB).

Results: In our research, we identified 21 children having multiple rare earth magnetic ingestions. The predominant symptoms were abdominal pain and vomiting which were observed in 57% (n = 12) and 48% (n = 10) of the patients respectively. The most common sign was abdominal tenderness,observed in 14% (n = 3) of the patients. In our sample, 38% (n = 8) of the patients were managed conservatively whereas 62% (n = 13) needed intervention. In our study, 48% (n = 10) of the patients sustained complications. The frequent complications were intestinal perforation appreciated in 24% (n = 5) and intestinal perforation with fistula formation in 19% (n = 4) of the patients. The median age of these patients was two years while the median number of magnets ingested was six. The ingestions were unwitnessed, and the duration of ingestions was unknowninthemajorityofpatientswhoexperiencedcomplications (n = 8/10).

Conclusion: If numerous rare earth magnetis ingested, children are in high danger of harm. It can be difficult to pinpoint the cases in younger children due to poor communication skills, especially if the intake is unreported. Although Qatar has established restrictions banning the import of rare earth magnets, there are reported cases of children with rare earth magnets ingestions.

## Introduction

Foreign body ingestion is common in children with a peak age of ingestion between 6 months and 3 years of age.^1^ Prevalently ingested foreign bodies are coins, batteries, toys, sharp objects, etc.^2^ The ingested foreign bodies are inclined to lodge at the narrow parts of the gastrointestinal tract i.e., cricopharyngeal part of the esophagus, middle one-third of the esophagus, lower esophageal sphincter, pylorus, and ileocecal valve. Of recent, there has been a rise in the ingestion of rare earth magnets by children. The esophagus is the most common location of the obstruction, but foreign things in the stomach and intestine typically pass on their own.^3,4^ Unlike traditional magnets, rare earth magnets are permanent magnets made from neodymium iron and boron and produce strong magnetic force. There is a risk of ingesting rare earth magnets used in several children's entertainment products.^5^ Rare earth magnets, when ingested in large quantities tend to cause complications such as intestinal perforation, fistula formation, intestinal obstruction, sepsis, and death.^6,7^ Our study's objective is to assess the clinical presentation and complications related to rare earth magnet ingestions in the pediatric population of Qatar.

## Methods

We carried out a retrospective chart review of children (ages between 0–14 years) who were presented to our Emergency Department at Sidra Medicine between January 2018 and July 2022 and were diagnosed as rare earth magnetic ingestions cases. We diagnosed 25 patients with rare earth magnet ingestions. This diagnosis was based on history, radiological findings, and operative notes. Of these 25 patients, four patients were excluded as they had single rare earth magnet ingestion. The remaining 21 patients with multiple rare earth magnetic ingestions were included in the study.

A standardized sheet was employed for data collection. This was an observational study,thus the personnel involved in data collection were not blinded. We obtained the data on age, gender, weight, vital signs, physical signs and symptoms, size of magnets, duration since ingestion, clinical management, and complications that occurred as a result of ingestions. (Table 1)

We employed descriptive statistics to calculate proportions for categorical variables. The 95 % confidence intervals were determined using SPSS software (IBM Corp, Armonk, NY) using the modified Wald technique. We applied median and interquartile range for continuous variables.

The institutional review board at Sidra Medicine on April 10, 2022, determined that our study qualifies for the exemption and continued oversight by IRB is unrequired. (IRB number: 1871284).

## Results

The median age of our sample size was 4 years (IQR 2–6) ranging from 1 to 10 years. Among our population, 12 (57%; 95% CI 34–78) were females and 9 (43%; 95% CI 22–66) were males.The median number of ingested magnets was 5 (IQR 2–9.5) ranging from 2 to 19. The median size of magnets was 5 mm (IQR 5.5–6). Magnetic ingestion was witnessed by the caregiver In thirteen patients (62%; 95% CI 38–82) and the median duration from the time of ingestion to the presentation at the emergency department was 4 hours (IQR 3–11). In eight patients (38%;18–62), the magnetic ingestion was unnoticed by the caregiver and the duration from ingestion to the presentation at the emergency department was uncertain.These patients presented to the hospital for assessment of abdominal pain and vomiting and the ingested magnets were revealed incidentally through radiographic imaging (X-rays).

The vital signs of the patients on arrival to the emergency department were median temperature: 36.9 (IQR 36.6–37), respiratory rate: 26 (IQR 24–28), heart rate: 101 (IQR 98–121), systolic blood pressure: 107 (IQR 99- 116) and diastolic blood pressure: 68 (IQR 58–77).

In our study, fourteen patients were symptomatic at presentation to the emergency department. The predominant symptom was abdominal pain followed by vomiting. Abdominal distention and tenderness were the most prevalent signs. Melena and dyspnea were not reported in any of the patients. (Table 1).

The past medical history was unremarkable in seventeen patients (83%; 95%CI 63–95), whereas 2 patients (13%, 95%CI 2–32) had a history of asthma, and each patient (4%; 95%CI 0–21) had autism and umbilical hernia respectively.

In our population, eight patients (38%; 18–62) were successfully managed conservatively with observation. None of them experienced any complications. The median age of these patients was 6 years (IQR 4.5–8.5). The median number of ingested magnets was 2 (IQR 2–6.5). All patients witnessed ingestion and the median duration of ingestion to the presentation at the emergency department was 3.5 hours (IQR 2.5–10).

The remaining thirteen patients 13 (62%; 38–82) needed invasive management in the form of endoscopy, laparoscopy, laparotomy, or combinations of these. Five of these required interventionsafter being initially treated conservatively due to either lack of progression on repeat imaging (cases 2,5,6,10; Table 2) or development of intestinal obstruction (case 4, Table 2). The remaining eight patients were taken to the operating room because of radiological and physical signs of intestinal obstruction (Cases 1,7,8,13; Table 2), perforation (Case 12; Table 2), location of magnets (magnets in the stomach, Case 3; Table 2 and magnets at the pharyngoepiglottic fold, Case 11; Table 2) and persistent symptoms such as vomiting and abdominal pain (Case 9; Table 2).

Ten patients in all experienced difficulties as a result of ingesting rare earth magnets. Among these, five patients (Cases 1,4,5,8 and 9; Table 2) sustained perforation of the bowel only, four patients (cases 7,10,12, and 13; Table 2) had bowel perforation associated with fistula formation, and one patient (Case 2; Table 2) had thinning of the intestinal mucosa.

The median age of patients who experienced complications (n = 10) was 2 years (IQR 1.5- 3). The median number of magnets ingested by these patients was 6 (IQR 4.5–16.5). Remarkably, the ingestion was unwitnessed in 8 patients, and the duration of ingestion was unknown. In 2 patients (cases 1,12; Table 2), the perforations were complicated by intra-abdominal abscess formation. The median duration of hospitalization for these patients was 11 days (IQR 6.5–13).

## Discussion

Over the past ten years, numerous nations throughout the world have reported rare earth magnets ingestion and the associated complications. Although it's uncommon for kids to consume rare earth magnets,their powerful magnetic field has been observed to cause severe gastrointestinal tract injuries.^8–10^ Therefore, as an emergency physician, it is critical to recognize the risks associated with rare earth magnetic ingestion.

Guidelines for the management of multiple magnet ingestion were released in 2015 by the North American Society for Pediatric Gastroenterology, Hepatology & Nutrition (NASPGHAN). If the magnets are in the stomach, they should be removed endoscopically, according to these recommendations. If the magnets are beyond the stomach and the patient is symptomatic, surgical interventions are needed. In asymptomatic patients, serial x-rays should be conducted to monitor progression, and in cases of lack of progression, surgical intervention should be performed.^11^ In our study, endoscopy was performed on 3 patients. In one patient (case 3; Table 2), an endoscopy was carried out, and magnets were extracted from the stomach without any complications. Conservative management was attempted initially for two days in the other two cases (cases 4,10; Table 2), but they developed signs of intestinal obstruction and perforations. In both cases, the magnets from the stomach were extracted endoscopically, and magnets from the intestine were removed via laparoscopy and laparotomy.

The rare earth magnets,commonly referred to as neodymium magnets, are 5–10 times more potent and are sold as small as 3–6 mm recreational objects.^12,13^ If more than one of these magnets is ingested or co-ingested with metallic objects, the intestinal wall is likely to become compressed, which can result in necrosis, perforation, fistula formation, obstruction, volvulus, sepsis, and death.^6,14^ In a multi-center study by Wang et al, perforation was found in 51% of their study population, while ischemia of the bowel was found in 19% of the population.^15^ Similar to a single-center study by Zheng et al., who reported single and multiple perforations in 37% of their study population, intestinal perforation was the most prevalent complication detected in our study with 43% of the study population.^16^

The rare earth magnets in toys are smaller in size and produce small perforations. In some cases, the formation of fistula and omentum sealing the small perforation results in mild symptoms and normal radiographic images. In our research, 2 patients (cases 5,9; Table 2) had mild symptoms with normal abdominal examination and normal radiographs (except magnets) but intraoperatively were discovered to have intestinal perforations. Similar cases of magnetic ingestions have been previously documented, in which thepatients either had mild symptoms or no symptoms, yet surgery revealed perforations and fistulas.^9,16,17^ Intestinal hemorrhage, hernia, volvulus, and death have also been linked to magnetic ingestions.^18–20^ In our study, none of the patients experienced these complications. In our study, the complications were noticed more often in toddlers who had unwitnessed ingestion. The tendency of toddlers to investigate their environment and accidentally swallow foreign objects can be used to explain this. The ingestions are also rarely suspected at this age because of weak communication skills, especially if they go unwitnessed.^15^

In 2012, US Consumer Product Safety Commission (CPSC) commenced regulatory activities to limit the sales of rare earth magnets followed by the implementation of a final rule in 2014, launching requirements for the sale of rare earth magnets.^21^ ED visits connected to magnet ingestions and hospitalization decreased as a result of the CPSP rule's effects.^22^ In 2017, this rule was overturned by the U.S. Court of Appeals for the Tenth Circuit.^23^ As a result, there were more ED visits associated with magnet ingestions and hospitalizations.According to Flaherty et al, from 2013–2016, the mean number of ED visits correlated to magnet ingestions reduced from 3.58–2.83 per 100,000 annually, followed by an increase to 5.16 per 100,000 annually after vacation of the CPSC rule.(13) CPSC implemented a new rule regarding the safety of rare earth magnets in September 2022. In this rule, CPSC has established acceptable criteria for the size and magnetic force of rare earth magnets sold as entertainment products.^24^ Rare earth magnetic toys were completely prohibited from entering Qatar as of September 2021. Intriguingly, despite the restriction, we still saw several pediatric patients with rare earth magnet ingestions. The reasons are uncertain and inquiry about them is out of the purview of this paper.

**Figure 1.**
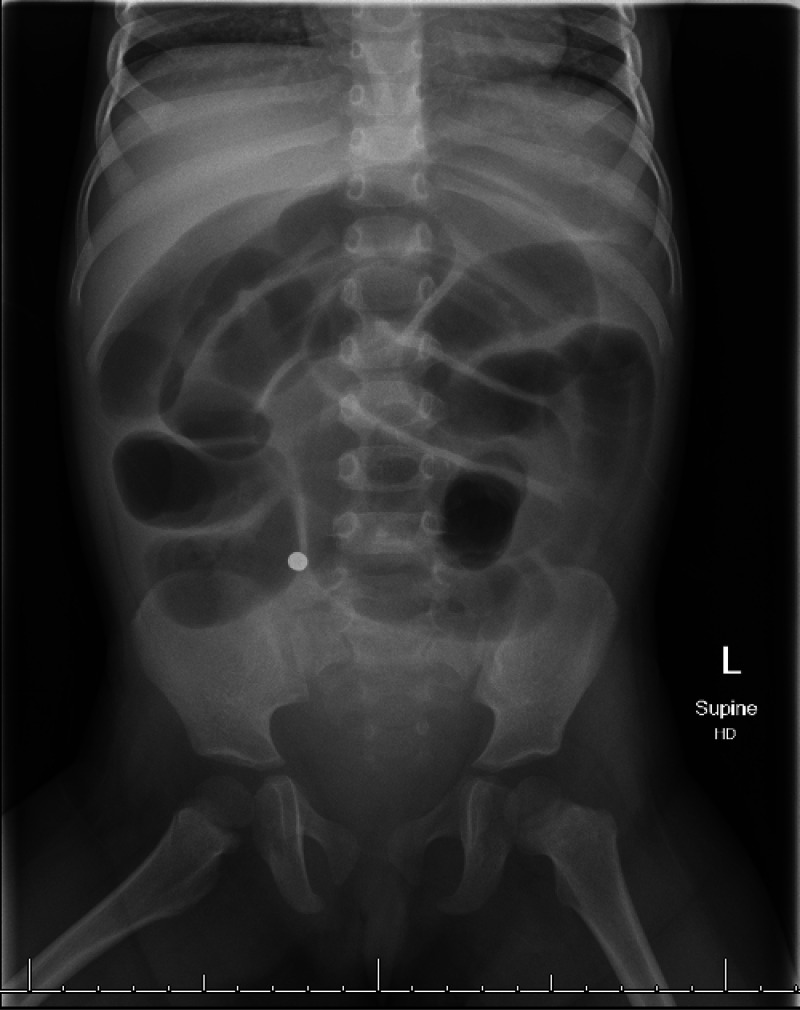
X-ray abdomen (frontal view), single rare earth magnet

**Figure 2.**
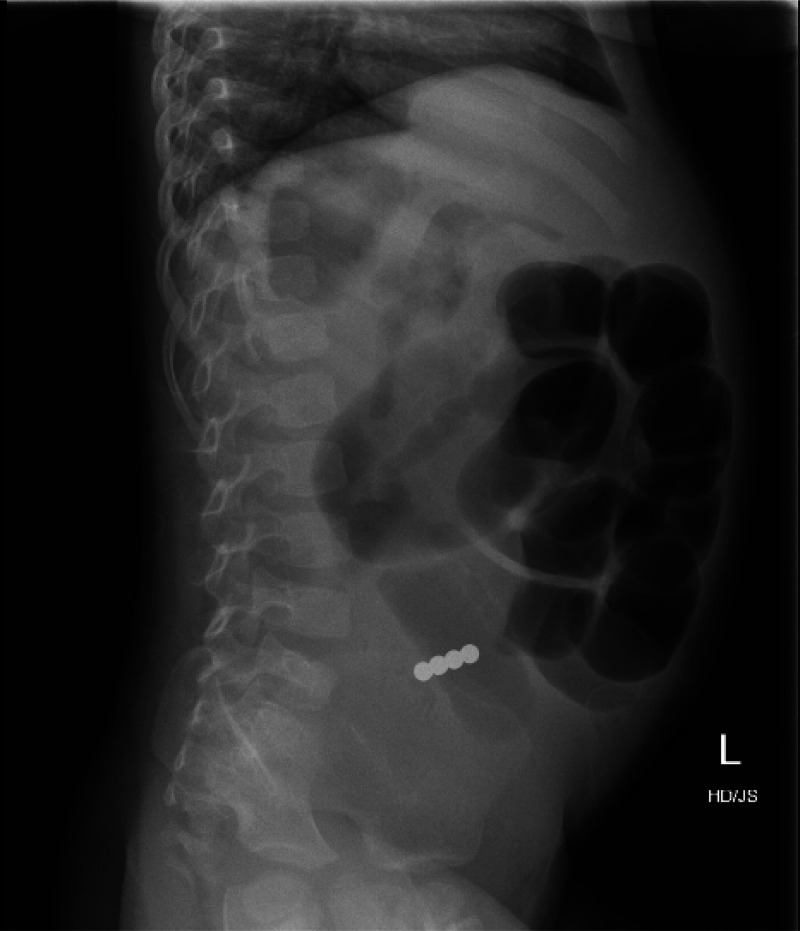
X-ray abdomen (lateral view), multiple rare earth magnets

Radiographs are crucial in the assessment of ingested foreign bodies.^25^ The images aid in identifying the location, size, shape, and number of foreign bodies ingested.^26^ To determine the precise location and several foreign bodies, it's crucial to get frontal and lateral radiographs.^12,27^ Single images can be misleading in identifying the true location and the number of foreign bodies. Hence, it is advisable to obtain “neck to rectum” x-rays in the evaluation of foreign bodies.^28^ In our research one patient (case 8; Table 2) seemed to have a single magnet on the initial frontal view whereas the lateral view indicated that 4 magnets clumped together. (Figures 1 and 2). In another patient (Case 11; Table 2) the initial chest radiograph overlooked the rare earth magnets in the pharynx which was later acknowledged on a neck x-ray involving a pharyngoepiglottic fold. (3)

## Limitations

This study has several limitations. First, since it is a retrospective chart review, it is conceivable that not all signs and symptoms were recorded for all patients. Therefore,our study possibly underreported the frequency of different signs and symptoms. Secondly, it is a single-center analysis of the emergency department. As a result, similar patients were likely treated at other institutions with different results. Thirdly, the outcomes are not generalizable to populations with different patient demographics because of the limited sample size.

## Conclusions

When consumed in large quantities, rare earth magnets can cause serious gastrointestinal injuries. In our study sample, approximately half of the patients (48%) who ingested rare earth magnets experienced complications. As unwitnessed ingestion is more likely to occur in children, the presence of rare earth magnets at home or in the vicinity of children should arouse suspicion of ingestion when gastrointestinal symptomspresent. Most of the individuals in our research sample who experienced difficulties were young children who had accidentally swallowed magnets. We propose raising public awareness about risks associated with the ingestion of rare earth magnets and education to keep the rare earth magnets away from the reach of young children.

To determine the precise number and location of ingested rare earth magnets, it is crucial to collect numerous views of radiographs during the investigation of magnet ingestions. In our study sample, occurrences of magnet ingestions also occur after the state of Qatar adopted regulations; this is a topic that requires more research. We also advise going over these rules again, and the US Consumer Product Safety Commission's new rules can provide additional guidance.

### Conflict of interest

We report no conflict of interest.

### Institutional review board determination

On April 10, 2022, the institutional review board at Sidra Medical and Research center determined that this study fits the exempt criteria. (IRB number: 1871284) Continuing review by IRB is not required.

## Figures and Tables

**Figure 3.**
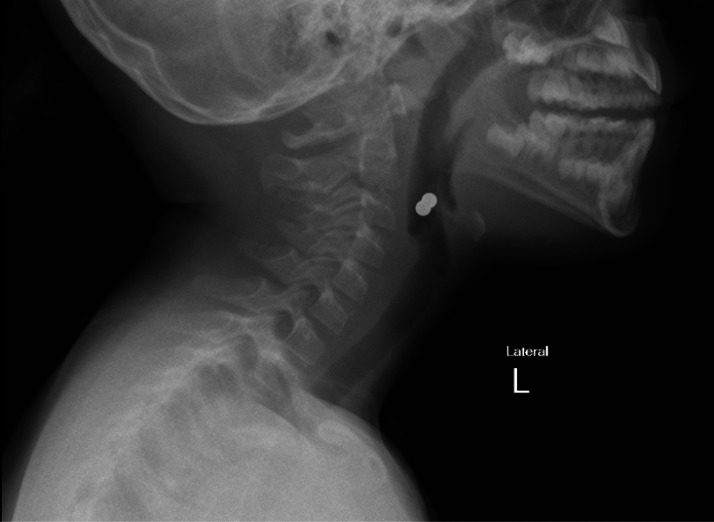
X-ray neck (lateral view), rare earth magnets clumped with pharyngoepiglottic fold

**Figure 4.**
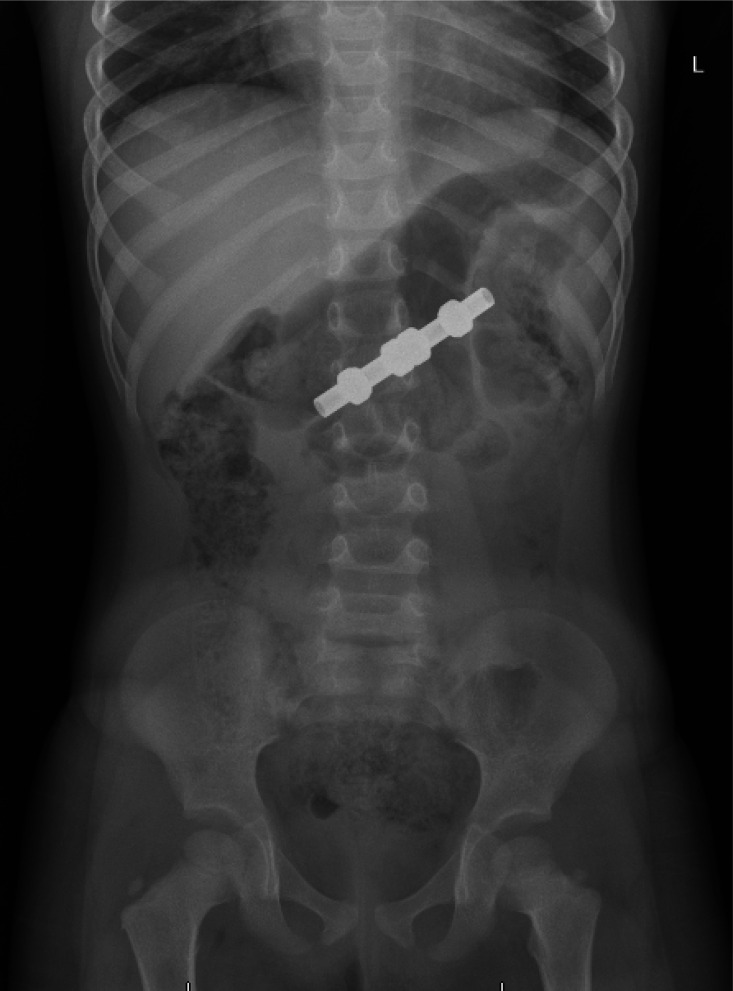
X ray abdomen (frontal view), dart magnets

**Table 1:** Clinical Characteristics of Children with Rare Earth Magnet Ingestions

Clinical features	Number, Frequency, and Confidence interval N (%; 95%CI)
Symptoms	14 (67%; 43–85)
Abdominal pain	12 (57%; 34–78)
Vomiting	10 (48%; 26–70)
Excessive crying	2 (9%; 1–30)
Cessation of bowel movement	2 (9%; 1–30)
Fever	1 (4%; 0–24)
Signs	2
• Abdominal distention	2 (9%; 1–30)
• Abdominal tenderness	3 (14%; 3–36)
Radiographs obtained	20 (100)
• Intestinal obstruction	4 (19%; 5–42)
• Pneumoperitoneum	1 (4%; 0–24)
Management	
• Conservative with success	8 (38%; 18–62)
• Intervention	13 (62%; 38–82)
○ Laryngoscopic removal	1 (5%; 0–24)
○ Endoscopy	1 (5%; 0–24)
○ Endoscopy plus surgery	2 (10%; 1–30)
○ Surgery (Laparoscopy and/or Laparotomy)	9 (43%; 23–66)
Complications	
• No complications	11 (52%; 30–74)
• Complications	10 (48%; 26–70)
○ Perforations	5 (24%; 8–27)
○ Perforations with fistula	4 (19%; 5–42)
○ Mucosal thinning	1 (4%; 0–24)

**Table 2:** Brief Description of Rare Earth Magnet Ingestion Cases Requiring Intervention

Cases	Age (yr)/Gender	Clinical presentation	Initial Imaging	Management	Intraoperative findings
1	1.5/M	Vomiting, abdominal pain for 2 days, and abdominal tenderness.Unwitnessed ingestion.	5 magnets in the left upper quadrant non-obstructive bowel gas pattern.	Laparoscopy plus laparotomy	Intra-abdominal pus 4 small bowel perforations and adhesion.
2	10/M	AsymptomaticWitnessed ingestions of magnets	6 magnets in the left upper quadrant.No abnormal bowel dilatation.No free air	Conservative initially followed by laparoscopic removal **due to lack of progression**	Bowel wall thinning but no perforations
3	4/M	Abdominal pain and vomiting for 1 day after witnessing ingestion	9 magnets in the stomach.Normal bowel gas pattern.No abnormal bowel dilatation.No free gas.	Upper GI endoscopy	3 pieces in the stomach 1 piece stuck across the mucosa of the gastroesophageal junction. No complications
4	3/F	Abdominal pain and vomiting for 1 day after swallowing 4 dart magnets. (Figure 4)	Foreign bodies in the mid-abdomen. No signs of bowel obstruction or free air.	Conservative for 2 days followed by upper GI endoscopy, laparoscopy plus laparotomy **due to intestinal obstruction**	3 pieces were extracted from the stomach. 4th piece from the colon Full-thickness colon perforation.
5	1.5/F	Abdominal pain, vomiting for 1 day.Unwitnessed ingestion.	A ring of magnets over the central abdomen.Non-obstructive bowel gas pattern.No free air	Conservative initially for 1 day followed by laparotomy **due to lack of progression**.	A 60 cm amalgamated small bowel loop with sealed antimesenteric perforation. 19 magnets removed
6	4/F	AsymptomaticAccidentally swallowed 2 magnets.	Magnets in the lower abdomen/pelvis. No pneumoperitoneum.	Conservative for 2 days followed by Laparoscopy **due to lack of progression**.	2 magnets were extracted from the cecum. No complications
7	2/M	Abdominal pain, vomiting, and abdominal tenderness for 1 day.Unwitnessed ingestion.	14 magnets projected over the right paravertebral region.Abnormal gaseous distension of small bowel. No free air	Laparoscopy plus laparotomy.	Dilated and hyperemic small bowel loops 3 perforations and enterocutaneous fistula
8	2/F	Vomiting, abdominal pain for 3 days, and distention for 1 dayUnwitnessed ingestion.	Magnets with distended small bowel The paucity of bowel gas in the distal colon and rectum	Laparotomy	Dilated small bowel 3 perforations in the terminal ileum and 1 perforation in the cecum
9	1.5/M	Recurrent vomiting and abdominal pain for 1 day Unwitnessed ingestions.	5 small, swallowed magnets in the medial right flank. non-obstructive bowel gas pattern. no evidence of pneumoperitoneum.	Laparoscopy plus laparotomy	4 pieces in the midileum with a peroration 5th piece in the duodenum without perforation
10	3/F	Abdominal pain and diarrhea for 1 day Unwitnessed ingestions.	Multiple magnets in the left side of the abdomen. The bowel gas pattern is nonspecific with no dilatation.	Conservative for 2 days followed by endoscopy and laparoscopy **due to lack of progression**.	6 magnetic beads stuck with gastric mucosa leading to superficial gastric fistula and perforation
11	6/F	Asymptomatic Accidentally swallowing 2 magnets.	Two magnets were identified in the neck at the level of the hypopharynx	Magnets were removed with Miguel forceps under general anesthesia.	Magnets stuck at the pharyngoepiglottic fold No complications
12	2/F	Vomiting, abdominal pain for 3 days, abdominal tenderness, and distention for 1 day. (2nd visit)Unwitnessed ingestion.	Multiple aggregated magnets in the lower abdomen/pelvis. A significant amount of free intraperitoneal air below both hemidiaphragm. Multiple dilated bowel loops with air-fluid levels.	Laparotomy.	19 magnets. ileo-ileal fistula. Gastro-jejunal fistula through the transverse mesocolon Intra-abdominal pus
13	1.5/F	Recurrent vomiting for 1 day.Unwitnessed ingestion.	Ten magnets were seen within the mid-abdomen. Multiple air-fluid levels were identified.	Laparotomy	5 magnets caused gastro-jejunal fistula and 5 magnets caused duodenal-jejunal fistula

## References

[1] Lee JH (2018). Foreign body ingestion in Children. Clin Endosc.

[2] Kay M, Wyllie R (2005). Pediatric foreign bodies and their management. Curr Gastroenterol Rep.

[3] Jayachandra S, Eslick GD (2013). A systematic review of paediatric foreign body ingestion: presentation, complications, and management. Int J Pediatr Otorhinolaryngol.

[4] Reeves PT, Rudolph B, Nylund CM (2020). Magnet ingestions in Children Presenting to Emergency Departments in The United States 2009–2019: a problem in flux. J Pediatr Gastroenterol Nutr.

[5] Hodges NL, Denny SA, Smith GA (2017). Rare-earth magnet ingestion-related injuries in the pediatric population: a review. Am J Lifestyle Med.

[6] (2006). MMWR Morb Mortal Wkly Rep.

[7] De Roo AC, Thompson MC, Chounthirath T, Xiang H, Cowles NA, Shmuylovskaya L (2013). Rare-earth magnet ingestion-related injuries among children, 2000-2012. Clin Pediatr.

[8] Sola R Jr, Rosenfeld EH, Yu YR, St Peter SD, Shah SR (2018). Magnet foreign body ingestion: rare occurrence but big consequences. J Pediatr Surg.

[9] Miyamoto R, Okuda M, Kaneko K, Numoto S, Okumura A (2019). Multiple magnets ingestion followed by intestinal fistula with mild symptoms. Glob Pediatr Health.

[10] Si X, Du B, Huang L (2016). Multiple magnetic foreign bodies causing severe digestive tract injuries in a child. Case Rep Gastroenterol.

[11] Kramer RE, Lerner DG, Lin T, Manfredi M, Shah M, Stephen TC (2015). Management of ingested foreign bodies in children: a clinical report of the NASPGHAN Endoscopy Committee. J Pediatr Gastroenterol Nutr.

[12] Hussain SZ, Bousvaros A, Gilger M, Mamula P, Gupta S, Kramer R (2012). Management of ingested magnets in children. J Pediatr Gastroenterol Nutr.

[13] Flaherty MR, Buchmiller T, Vangel M, Lee LK (2020). Pediatric magnet ingestions after federal rule changes, 2009–2019. JAMA.

[14] Agbo C, Lee L, Chiang V, Landscahft A, Kimia T, Monuteaux MC, et al (2013). Magnet-related injury rates in children: a single hospital experience. J Pediatr Gastroenterol Nutr.

[15] Wang K, Zhang D, Li X, Wang Z, Hou G, Jia X (2020). Multicenter investigation of pediatric gastrointestinal tract magnets ingestion in China. BMC Pediatr.

[16] Zheng Y, Zhang Z, Yan K, Guo H, Li M, Lian M (2021). Retrospective analysis of pediatric patients with multiple rare-earth magnets ingestion: a single-center experience from China. BMC Pediatr.

[17] Taher H, Azzam A, Khowailed O, Elseoudi M, Shaban M, Eltagy G (2019). A case report of an asymptomatic male child with multiple entero-enteric fistulae post multiple magnet ingestion. Int J Surg Case Rep.

[18] Waters AM, Teitelbaum DH, Thorne V, Bousvaros A, Noel RA, Beierle EA (2015). Surgical management and morbidity of pediatric magnet ingestions. J Surg Res.

[19] Bauman B, McEachron K, Goldman D, Louiselle A, Zheng E, Mills D (2019). Emergency management of the ingested magnet: an algorithmic approach. Pediatr Emerg Care.

[20] Nui A, Hirama T, Katsuramaki T, Maeda T, Meguro M, Nagayama M (2005). An intestinal volvulus caused by multiple magnet ingestion: an unexpected risk in children. J Pediatr Surg.

[21] http://.

[22] Reeves PT, Nylund CM, Krishnamurthy J, Noel RA, Abbas MI (2018). Trends of magnet ingestion in children, an ironic attraction. J Pediatr Gastroenterol Nutr.

[23] https://www.federalregister.gov/d/2017-04381.

[24] https://www.cpsc.gov/newsroom/news-releases/2022/cpsc-approves-new-federal-safety-standard-for-magnets-to-prevent-deaths-and-serious-injuries-from-high-powered-magnet-ingestion.

[25] Hodge D 3rd, Tecklenburg F, Fleisher G (1985). Coin ingestion: does every child need a radiograph?. Ann Emerg Med.

[26] (2011). Gastrointest Endosc.

[27] Guelfguat M, Kaplinskiy V, Reddy SH, DiPoce J (2014). Clinical guidelines for imaging and reporting ingested foreign bodies. AJR Am J Roentgenol.

[28] Tseng HJ, Hanna TN, Shuaib W, Aized M, Khosa F, Linnau KF (2015). Imaging foreign bodies: Ingested, aspirated, and inserted. Ann Emerg Med.

